# View point: gaps in the current guidelines for the prevention of Methicillin-resistant *Staphylococcus aureus* surgical site infections

**DOI:** 10.1186/s13756-018-0407-0

**Published:** 2018-09-18

**Authors:** Kevin T. Kavanagh, Said Abusalem, Lindsay E. Calderon

**Affiliations:** 1 Health Watch USA, Somerset, KY USA; 2 Health Watch USA, University of Louisville, Louisville, KY USA; 3 Health Watch USA, Eastern Kentucky University, Richmond, KY USA

## Abstract

The authors advocate the addition of two preventative strategies to the current United State’s guidelines for the prevention of surgical site infections. It is known that *Staphylococcus aureus*, including Methicillin-resistant *Staphylococcus aureus* (MRSA), carriers are at a higher risk for the development of infections and they can easily transmit the organism. The carriage rate of *Staph. aureus* in the general population approximates 33%. The CDC estimates the carriage rate of MRSA in the United States is approximately 2%. The first strategy is preoperative screening of surgical patients for *Staph. aureus*, including MRSA. This recommendation is based upon the growing literature which shows a benefit in both prevention of infections and guidance in preoperative antibiotic selection. The second is performing MRSA active surveillance screening on healthcare workers. The carriage rate of MRSA in healthcare workers approximates 5% and there are concerns of transmission of this pathogen to patients. MRSA decolonization of healthcare workers has been reported to approach a success rate of 90%. Healthcare workers colonized with dangerous pathogens, including MRSA, should be assigned to non-patient contact work areas. In addition, there needs to be implemented a safety net for both the worker’s economic security and healthcare. Finally, a reporting system for the healthcare worker acquisition and infections with dangerous pathogens needs to be implemented. These recommendations are needed because *Staph. aureus* including MRSA is endemic in the United States. Policies regarding endemic pathogens which are to be implemented only upon the occurrence of a facility defined “outbreak” have to be questioned, since absence of infections does not mean absence of transmission. Optimizing these policies will require further research but until then we should error on the side of patient safety.

## Commentary

The emergence of multi-drug resistant organisms presents new challenges for the prevention of surgical site infections (SSI). In July of 2017, the CDC issued additional guidance on preventative strategies [1]. However, there remain a number of important gaps in the prevention of spread of Methicillin-resistant *Staphylococcus aureus* (MRSA) and other multi-drug resistant drug organisms.

There is mounting evidence regarding the importance of assessing the patient’s microbiome for improved treatments and prevention of hospital acquired infections [2]. In the future, characterizing a patient’s commensal and pathogenic bacteria may become common place and practical from both an economic and logistical standpoint. Presently, there is readily available technology to at least screen patients and healthcare workers for dangerous pathogens.

Two additional standards should be adopted to prevent SSI: 1) Preoperative screening and decolonization for *Staph. aureus*, including MRSA in patients and 2) screening and decolonization of MRSA in healthcare workers, along with the implementation of an event reporting system and the development of protocols for financial and healthcare protection.

### Screening of *Staph. aureus* to prevent surgical site infections

*Staph. aureus* is a common pathogen which causes post-operative infections [3]. It has been demonstrated that between 26 to 37% of patients are *Staph. aureus* carriers [4–6] and that these carriers have higher rates of SSIs [3, 7]. Decolonization has been shown to be effective in decreasing *Staph aureus* SSIs [3, 4, 8].

Although not widely performed in the United States, WHO has recently recommended decolonization of *Staph. aureus* carriers for the prevention of SSI, with preoperative patient screening to implement this intervention. This is a strong recommendation for cardiothoracic and orthopedic surgery and a moderate conditional recommendation “when feasible” because of costs for other types of surgery [3]. Similar to the United Kingdom’s National Health Service, [9] the United States has the financial resources to implement this policy on all surgical patients.

### Screening of MRSA to prevent surgical site infections

In the United States, screening is not routinely performed for *Staph. aureus* in preoperative patients, and there has even been reluctance to screen for the more resistant form of *Staph. aureus*, MRSA. A major concern regarding MRSA is the continued progression and spread of antibiotic resistance, with additional resistance developing to vancomycin and other antibiotics. In addition, there is mounting evidence regarding increase virulence through genetic acquisition and expression [10, 11]. However, there has been abundant research regarding the mechanisms of spread and strategies to control the spread of MRSA, along with analysis of the resistance to setting firm standards to stop this epidemic.

In the United States, screening for MRSA fell out of popularity in part due to a study published in JAMA by Harbarth, et al. [12]. However, this study was poorly implemented as antibiotics active against MRSA were not administered to 57% of patients known to be MRSA carriers preoperatively (30% of the total number of patients). In 31% of the total patients, the MRSA cultures were not available until after the surgery [12]. However, the data also indicated that MRSA carriers were over 14 times more likely to develop a postoperative MRSA infection than non-carriers (Chi Squire with Yates Correction, *p* < 0.0001).

Since, the study by Harbarth, et al. [12] was published, Kavanagh, et al. [13] reviewed 19 studies that indicated a beneficial effect of preoperative MRSA screening, 14 studies reached statistical significance. A wide range of patients were studied including patients in intensive care, and those undergoing vascular, orthopedic, gynecologic, gastrostomy, head and neck, and cardiac surgeries. The vast majority of these studies used pre-post designs and had non-concurrent controls; however, the large number of studies that showed positive effects of pre-operative MRSA screening outweighs the effects of random unknown variables in the pre-post studies [13].

Preoperative knowledge of the MRSA status of surgical patients allows for both the proper selection of preoperative antibiotics and in elective surgical patients, implementation of a preoperative decolonization protocol. MRSA carriers are often prophylaxed with vancomycin or teicoplanin as opposed to a cephalosporin. There is evidence that the use of vancomycin in non-MRSA carriers may lead to an increase in post-surgical infections [14]. In addition, Branch-Elliman, et al., concluded that vancomycin usage in cardiac patients was associated with an increased risk of acute kidney injury and concluded that its risks in MRSA negative patients outweighs its benefits [15].

The following studies have focused on MRSA and support the policies for screening for this pathogen:

- MRSA is highly infectious, and carriers have been observed to spread the organism to the environment at a higher rate than those with infections [16].
- It has been observed that 35% of MRSA colonized patients can contaminate their environment within 33 h [17] and once contaminated, surfaces may be MRSA culture positive for up to one to two months [18].
- The CDC estimates that the United States’ general population has a carrier rate of 2% [19].
- Multiple studies found a higher carrier rate in healthcare workers. On average, the rate approaches 5% [20, 21].
- Albrich and Harbarth found 79 studies that support the spread of MRSA from healthcare workers to patients [20].
- MRSA carriers are at an increased risk for MRSA infections [22].
- The fatality rate for MSA bloodstream infections approximates 16% [23].

In the United States, MRSA bloodstream infections are not on track to meeting the 2020 goals of a 50% reduction, there is some evidence that in 2015 these infections may have even increased [24].

### Transmission of staph aureus and MRSA from healthcare workers to patients

MRSA is endemic in the United States. The CDC estimates that 2% of the general population are carriers [19] and according to the CDDEP 43% of outpatient *Staph. aureus* cultures are oxacillin resistant (2012 data) [25]. So why has there not been more research directed at healthcare worker colonization?

It has been demonstrated that healthcare workers can transmit *Staph. aureus* to patients. Price JR, et al. demonstrated that out of 25 instances of patient acquisition of *Staph. aureus*, seven were from healthcare workers [5]. Albrich and Harbarth reviewed 68 articles which performed genotyping on colonized and infected healthcare workers and found that 93% had likely transmission from healthcare worker to patient [20]. And, at least one study has reported a decrease in the incidence of MRSA with the identification and treatment of healthcare workers [26].

There is also no reason to believe that transmission of MRSA will differ between different groups of individuals and would be expected to carry equal risks in the general public, patients and healthcare workers. Cadena, et al. observed that patients who are MRSA carriers and are diabetics, on dialysis, or undergoing surgery are at a higher risk for developing MRSA infections than non-carriers [22]. Unlike, the general public, healthcare workers come into contact with some of the most frail and susceptible individuals and thus, prevention of spread should be imperative.

### Protocols for screening and decolonization

The protocol commonly used for *Staph. aureus* decolonization is daily body washes with an antiseptic for five days plus an intranasal antibiotic ointment, given three times a day. For MRSA positive patients, 2% mupirocin is often applied intranasally; for MSSA positive patients, Naseptin Nasal Cream (chlorhexidine hydrochloride 0.1%; neomycin sulfate 0.5%) can be used [27]. Eradication of the MRSA carrier state was achieved in up to 88% of healthcare workers [20]. Universal decolonization of all patients or healthcare personnel regardless of their carrier state should be avoided because of the potential to spread resistance [3, 28].

Similar to patients, the healthcare worker poses a risk of spreading pathogens to the environment. Healthcare employers should not assign healthcare workers in patient contact areas while they are carriers of dangerous pathogens. We feel this policy should apply to MRSA carriers not only because of the pathogen’s resistance to antibiotics but also because of concerns regarding increase virulence [3, 10, 11].

### Health policy decisions

During the July 2017 Centers for Disease Control and Prevention’s Healthcare Infection Control Practices Advisory Committee (HICPAC) meeting, the committee discussed the topic of MRSA control as it relates to healthcare workers. Under consideration was a recommendation to screen healthcare workers during an MRSA outbreak.

#### Endemic or outbreak

The occurrence of an “Outbreak” is often used to trigger increased infection control and prevention protocols. However, in the United States, an “outbreak” is often defined as a rate of infections above a facility defined baseline. Thus, daily MRSA infections could theoretically be occurring and neither patients, the health department or oversight agencies would be notified of an outbreak in the facility. It all depends upon how many infections it takes to be greater than the facility defined baseline.

Thus, we should not guide our actions around the non-standardized term of “outbreak”. We agree with Albrich and Harbarth [20] whose findings support not restricting screening of MRSA to outbreaks in endemic settings.

There is little doubt that MRSA has become endemic in the United States. The absence of an outbreak does not mean there is absence of exposure or transmission; it does not even mean there have been no infections. Our perspective on MRSA and interventions to mitigate it, need to undergo a transformative paradigm shift.

#### The healthcare worker

Many categories of healthcare workers contact patients on multiple types of wards. In addition, the increasing trend of “cross-training” of nurses and the use of temporary nurses to save costs, results in the same personnel being assigned to different types of wards. Thus, policies relating to healthcare workers should be facility wide.

The reason for not adopting protocols for readily identifying and controlling pathogen in healthcare workers may lie in conflict-of-interest in the healthcare industry. Detection and decolonization may have a negative financial impact on institutions. All too often, the healthcare worker is put at risk because well-defined occupational safety and healthcare standards for infection control have not been established. Part of this dilemma is the lack of research. By not investigating, a call to action regarding the healthcare workers, along with the health of patients, has been severely mitigated and the development of effective protocols to reduce post-surgical infection rates have been hampered. At the same time difficult and costly decisions have been avoided.

In addition, the United States does not have a national surveillance system for the tracking of healthcare worker acquisition and infections with dangerous pathogens. This results in cases not being recorded and classified as work related. Healthcare staff will often use sick leave or work while ill. Additionally, there is a concern regarding healthcare workers bringing antibiotic resistant pathogens home to their families. Once MRSA is transmitted into a household environment, without further intervention, it may persist for 2.33 to 8.55 years [29].

#### Need for better standards

The lack of effective standards for the protection of healthcare workers is not restricted to MRSA. The Ebola epidemic underscored the inadequacy of hospital preparedness to deal with dangerous pathogens. On Aug 7, 2014, assurances were given to the U.S. House Foreign Affairs Subcommittee that healthcare facilities in the United States are trained in and had protocols in place to handle Ebola [30]. However, by October of that year, two healthcare workers became infected and it became evident that safety protocols were inadequate and that only a few special biocontainment facilities in the United States could handle patients with highly contagious diseases.

Although MRSA is not as deadly as Ebola, it is highly infectious. According to the Centers of Disease Control and Prevention (CDC), the number of deaths in the United States from MRSA is at least 11,000 each year and there are over 80,000 severe infections [31]. These statistics demonstrate the urgency of improving practices for the protection of patients and staff from MRSA and other multi-drug resistant pathogens. The hospital cost of treating MRSA patients is between $3.2 and $4.2 billion [32]. This expenditure demonstrates that improved infection prevention strategies, such as those proposed in this paper, can have direct healthcare dollar savings plus improve performance and reimbursement with CMS’s value-based purchasing initiatives.

#### Policy recommendations

The following policies should be enacted:

1. The screening of patients and healthcare workers for dangerous pathogens, including MRSA, to mitigate the changes of spread to other patients, staff and the community.
2. Establish a surveillance system for healthcare workers to determine the extent of occupational acquisition and infections of dangerous pathogens, including MRSA.
3. Establish an economic and healthcare safety net for healthcare workers who become infected or colonized with dangerous pathogens, including MRSA.
4. Research to promote effective screening and decolonization of healthcare workers for dangerous pathogens, including MRSA.
5. Assessing the effectiveness of the diverse current practices in the healthcare industry in order to implement standardized prevention strategies, including screening protocols. For example, the comparing of the results of the MRSA prevention protocols in the U.S. Veterans Health Administration's healthcare system with those of private industry [24].

In the United States, increased vigilance is necessary. Data is not available for many types of infections, but for MRSA bloodstream infections the data indicates that they may not be coming under control. Data from the National Healthcare Safety Network indicated that MRSA infections increased between 2014 to 2015 and that we are not on track to meet the national goal of a 50% reduction in MRSA bloodstream infections by 2020 [24].

## Conclusion

Many now believe that knowledge of a patient’s microbiome will one day be essential for the maintenance of good health and may even become a part of a yearly physical exam. Until resources are available to accomplish this, we should at a minimum initiative screening of surgical patients for *Staph. aureus*, including MRSA. Similar to the United Kingdom’s National Health Service, [9] the United States has the resources to implement the WHO recommendations [3] regarding the screening of all surgical patients for *Staph. aureus*.

The United States also need to implement programs for periodic screening of all healthcare workers, including surgical staff, for the resistant form of *Staph. aureus*, MRSA. Healthcare workers who are positive should be decolonize and whenever possible assigned to nonpatient contact areas until decolonization is complete. Decolonization of healthcare workers is highly successful with 88% MRSA eradication [20]. Work reassignment programs need to protect affected worker's job and income status and should be an essential component of the policy.

The gaps we have addressed deserve more research including the risks a colonized healthcare worker poses to patients, the number of healthcare workers exposed and infected occupationally, and the development of effective healthcare worker occupational infectious disease surveillance programs. Nevertheless, current evidence indicates that colonization with dangerous pathogens is a critical issue which has the potential of causing grave harm. The existence of research gaps does not mean we should not act, because not acting is in itself an action. It is our duty to deliver patient centered care of the highest quality and until we know a practice, or an inaction is safe, we must error on the side of patient and healthcare worker safety.
